# Risk factors for subdural hematomas in patients shunted for idiopathic normal pressure hydrocephalus

**DOI:** 10.1186/2045-8118-12-S1-O47

**Published:** 2015-09-18

**Authors:** Nina Sundström, Johan Wallmark, Anders Eklund, Lars-Owe Koskinen, Jan Malm

**Affiliations:** 1 Department of Radiation Sciences, Biomedical Engineering, Umeå University, Umeå, Sweden; 2 Department of Clinical Neuroscience, Umeå University, Umeå, Sweden; 3 Department of Neurosurgery, Division of Pharmacology and Clinical Neuroscience, Umeå University, Umeå, Sweden

## Introduction

Subdural hematoma (SDH) is a common complication of shunt surgery, but it is not clear which are the most important underlying causes. Based on a large prospective population based material, the objective of this study was to identify the most important risk factors for postsurgical SDH in patients with idiopathic normal pressure hydrocephalus (iNPH).

## Methods

The Swedish Hydrocephalus Quality Registry (SHQR) is a prospective population based database in which hydrocephalus surgeries have been recorded nationwide since 2004. In this study, 1458 iNPH patients shunted between 2004 and 2014 were included. Post surgery, 122 (8.4 %) developed SDH. The prevalence of risk factors for SDH was compared between patients with and without postoperative SDH (controls).

## Results

Median time between surgery and SDH was 125 days. Men were predisposed for development of SDH (73 % males in the SDH group vs. 59.6 % in the control group, p=0.004). Initial, as well as last shunt opening pressure (Popen) before SDH were lower in the SDH group compared to the controls (108.0 vs. 115.5 mmH2O (p=0.003) and 98.8 vs. 107.5 mmH2O (p=0.006) respectively). Antisiphoning devices (ASD) were used to the same extent in both groups. On average, Popen was 21.7 and 24.6 mmH2O lower in shunts with than without an ASD at time of surgery and last adjustment respectively. Despite this, the proportion of SDHs was the same in both groups. No differences were seen for adjustable vs. fixed valves, proximal catheter location, ASA physical status classification system score, Rankin scale score or co-morbidity.

## Conclusions

Despite new shunt designs, SDH is still a common complication in patients shunted for iNPH. The predominant risk factors were found to be male sex and lower Popen. The complication was also related to the presence or absence of an ASD, since an ASD allowed for a lower Popen without an increased complication rate. This indicates that hydrodynamical issues play an important role in the development of SDH in iNPH patients. Thus, methods allowing individual adjustment of Popen are needed in order to optimize functional improvement and minimize the number of SDHs.

